# Parental Burnout and Prosocial Behavior among Chinese Adolescents: The Role of Empathy

**DOI:** 10.3390/bs14010017

**Published:** 2023-12-25

**Authors:** Qichen Wang, Yue Lin, Ziwen Teuber, Fangmin Li, Yanjie Su

**Affiliations:** 1 Beijing Key Laboratory of Behavior and Mental Health, School of Psychological and Cognitive Sciences, Peking University, No. 5 Yiheyuan, Haidian, Beijing 100871, China; wangqichen628@pku.edu.cn (Q.W.); linyue_psy@stu.pku.edu.cn (Y.L.); fangminli@pku.edu.cn (F.L.); 2 Department of Psychology, Bielefeld University, Universitaetsstr. 25, 33615 Bielefeld, Germany

**Keywords:** adolescents, parental burnout, prosocial behavior, empathy, parent–children interaction, sequential mediation, socialization

## Abstract

Parental burnout refers to exhaustion caused by the parenting role. This devastating negative emotion can have repercussions for adolescent social development. Nevertheless, much remains unclear about the association between parental burnout and adolescent prosocial behavior and the potential mechanisms underlying this relationship. Based on theoretical and empirical evidence, the current study examined the relationship between parental burnout and adolescent prosocial behavior by using a sequential mediation model that included both parental empathy and adolescent empathy as potential mediators. A total of 488 parent–adolescent dyads (for adolescents: 45.7% men, 54.3% women, *M*_age_ = 15.28 ± 1.67 years; for parents: 36.5% fathers, 63.5% mothers, *M*_age_ = 41.30 ± 3.79 years) completed questionnaires regarding demographics, social desirability, parental burnout, parental empathy, adolescent empathy, and adolescent prosocial behavior. After controlling for demographic covariates and social desirability, the results showed that parental burnout had a negative effect on adolescent-reported prosocial behavior through parental cognitive empathy and adolescent other-oriented empathy (adolescent cognitive empathy and empathic concern) sequentially. These findings contribute to our understanding of the role of parental burnout as a family environmental factor detrimental to the positive functioning of adolescents through parental reactions to their children’s emotions and children’s own social competence.

## 1. Introduction

Prosocial behavior, a result of an individual’s socialization, is defined as voluntary actions aimed at assisting or benefiting others, such as cooperating, sharing, and consoling others [1]. It not only improves the recipients’ well-being but also contributes to the development of the helper by improving physical health [2,3], alleviating negative states [4], and promoting interpersonal connections [5]. Adolescence is a critical stage of individual emotional socialization and prosocial development [6]. Adolescents showing prosocial behaviors or higher prosocial tendencies perform better in terms of academic and social adjustment, and this effect persists into adulthood [7].

Family is an important place for individual socialization [8]. Parental factors have a significant influence on both adolescent socialization and prosocial behavior [9]. Parental burnout results from a long-term imbalance between overwhelming stressors and coping resources [10]. It has recently received increased attention worldwide [11,12] and may have a negative influence on parental interactions with their children [10] and their children’s behavioral outcomes [13]. Adolescence is a period in which the frequency and intensity of parent–child conflicts are increasing because of the adolescent children’s characteristic lability and irritability [14]. As a result, the adolescents’ parents may find parenting practices more challenging and perceive much more stress [15]. Experiencing challenges and stress over time will increase their susceptibility to parental burnout. However, previous studies have ignored the relationship between parental burnout and adolescent positive behavioral outcomes. Thus, we examined the effect of parental burnout on adolescents’ prosocial behavior and whether parental empathy and adolescent empathy were potential mediators of this relationship.

### 1.1. The Potential Mediating Role of Parental Empathy on Their Child

Parental burnout is typically characterized by parents’ exhausted feeling in terms of their parenting role or sudden negative changes in their parental attitude [11]. While existing research has mainly focused on the antecedent variance of this phenomenology, such as socially prescribed perfectionism, high neuroticism, and low conscientiousness [16,17], and its negative impacts on parents themselves (e.g., depression and sleep disorders) [18,19] as well as their parenting behaviors (e.g., parents’ escape ideation, child neglect, and violence) [10,20,21], whether parental burnout impairs the positive development outcomes of adolescents and the pathway by which the impairment takes place remain unclear.

Theoretical studies suggest that parenting burnout may have a negative effect on adolescent prosocial behavior. As mentioned above, prosocial behavior is the result of individual socialization, which is the process whereby an individual learns knowledge, norms, and other social behaviors in a specific sociocultural environment [22]. Prosocial behavior, to a large extent, depends on one’s understanding of other’s emotions and the causes behind them in social interactions [23]. According to the theoretical model of the socialization of emotion, parents’ own emotions and their regulation are critical to a child’s emotion socialization and can affect their responses to children’s emotions, which in turn is related to the emotional competence and social behavior of the children [8,24]. Parental burnout is a comprehensive indicator of long-term perceptions of stress or negative emotions and failure to regulate, which leads parents to escape from their children when they are in need [20,21]. As a prosocial scaffold, appropriate parent–child interactions model the way their children should behave kindly toward others who need help [8,24]. On the contrary, maladaptive interactions due to parental burnout may be negatively related to adolescent prosocial behavior.

In line with the mentioned theory, there are some indirect empirical results indicating the negative relationship between parental burnout and adolescent prosocial behavior. For example, children with anxious parents are less other-oriented and show more avoidant behavior [25]. In addition, prior studies have shown that parenting stress, a main cause of parental burnout, had longitudinally negative repercussions on adolescents’ prosocial behavior [26,27]. Most of these studies were conducted in Western countries among adolescents and their parents. Moreover, a longitudinal study in China has also shown that Chinese parental burnout leads to more negative behavioral outcomes, like the externalized problems of adolescents [28]. Although the relationship between parental burnout and adolescent prosocial behavior has been supported by some theoretical and indirect empirical evidence, no direct empirical studies so far have investigated the relationship between them. Moreover, the theoretical study mentioned above indicates that the relationship between parental burnout and adolescent behavioral outcomes may not be that simple [8,10,24]. Parental empathy, which indicates how parents react to their children’s emotions, may be one of the potential mediators [8,24].

From a theoretical perspective, parental cognitive empathy may be positively related to adolescent prosocial behavior. Parental empathy refers to the parental empathic reactions to their child’s emotions. It can be further divided into parental cognitive empathy (child-oriented reactions, indicate the understanding of the child’s emotions and the causes or needs behind them) and parental affective empathy (parental self-oriented emotional contagion reactions, such as feelings of distress or anxiety in the face of the child’s negative emotions) [29]. Prosocial behavior is other-oriented and requires an understanding of targets’ emotions and the needs behind them [30]. Traced back to the socialization of emotion model, children react to others’ emotions and needs in the way that they are treated by their parents in a family context [8,24]. Accordingly, parents with higher parental cognitive empathy understand their children’s emotions and needs accurately. They will set a prosocial example for their children by performing more child-oriented supporting behaviors. However, parents who share more emotions, especially negative emotions with their children, always fail to get rid of their own vicarious negative emotions. Therefore, they may have difficulty in providing supportive behavior when their children need it and modeling prosocial behavior for their children [31].

In line with theoretical explanations, previous empirical findings also indicate that two facets of parental empathy may function differently from children’s behavioral outcomes. One of the studies found that Chinese children with higher-parental-cognitive-empathy parents showed less problematic behavior [32], while another study that used a total parental empathy score did not find any significant relationship between Chinese parental empathy and their children’s adjustment [33]. The mixed results suggest that differentiating between two facts of parental empathy toward their children is necessary. And, parental cognitive empathy may be a positive predictor of adolescent prosocial behavior.

Unfortunately, parental cognitive empathy could be deteriorated by parental burnout, and this has been supported by both theory and empirical results. From a theoretical point of view, parental empathy, reflecting parental reactions to their child’s emotions, may be a result of the parents’ own emotional state (i.e., parental burnout). Empirical studies also provide some hints for hypotheses formulation. As a top-down process of the children’s emotions or experiences, parental cognitive empathy needs cognitive resources [34], whereas the chronic and overwhelming stress of burned-out parents and the related physical suffering, such as an impaired quality of sleep [35], would reduce the resources for cognitive empathy [36]. In addition, a longitudinal study showed that mothers who experience parental burnout showed less supporting and even more hostile and violent behavior toward their children [13,28]. Taking that child abuse is an indicator of lacking parental cognitive empathy, which has been shown in a parent–child matching study [37], parental burnout may be negatively related to parental cognitive empathy. Since most of the mentioned empirical works were conducted in Western countries, addressing this question in a Chinese context is necessary. 

Based on the above-presented evidence, it is reasonable to make the first hypothesis as follows:

**H1.**  *Parental burnout would negatively predict adolescent prosocial behavior through parental cognitive empathy.*

### 1.2. The Potential Mediating Role of Adolescent Empathy Ability in Sequential Pathway

One’s empathy ability indicates their social competence to share and understand the emotional states of others. As an umbrella term, it can be further divided into three components, namely affective empathy (entails vicariously experiencing the targets’ emotion), cognitive empathy (entails the ability to represent what the targets feel and why), and empathic concern (the motivation to improve the target’s well-being without necessarily taking on his emotional states) [34,38,39]. Among them, the affective component, undoubtedly, is the self-oriented component of empathy, whereas cognitive empathy and empathic concern are self-oriented components that may serve as positive predictors of prosocial development [34].

Theoretically, as stated by the empathy–altruism hypothesis [30], prosocial motivation is elicited by empathy. However, a recent theoretical study expands this mentioned theory by distinguishing the functions of different empathic components [34]. Individuals with higher other-oriented empathy have more attentional resources to concentrate on the target’s feelings and perform prosocial behaviors by accurately analyzing the target’s needs [31,38].

Those theory works have been supported by the following empirical findings. For instance, a systematic review of longitudinal studies found that adolescent cognitive empathy, as measured by both behavioral experiments and self-reported scales, stably predicted their prosocial behavior [40]. Similarly, longitudinal studies show that empathic concern, another component of other-oriented empathy, is a stable predictor of Italian adolescent prosocial behavior [41], while for self-oriented empathy, the result was the opposite. Empirical evidence suggests that American adolescent affective empathy does not have a significant or even negative relationship with prosocial behaviors [42]. The reason for this could be that individuals with higher affective empathy may vicariously take on the target’s negative affect in prosocial situations and then stay away from the distressed target to avoid uncomfortable feelings [31,38]. Because of adolescents’ sensitivity to others’ emotional cues, the mentioned avoidance mechanism, caused by affective empathy, is particularly strong in adolescence [43]. Collectively, adolescent other-oriented empathy, namely cognitive empathy and empathic concern, may be positively related to prosocial behaviors.

Adolescent other-oriented empathy, a potential fostering factor of prosocial behavior, may be affected by parental burnout and parental cognitive empathy. According to the theoretical model of the socialization of emotion, a child’s social competence to deal with the emotions of others reflects how their own emotions have been addressed by their parents in a family context [8,24]. Unlike affective empathy, which matures early in life, other-oriented empathy continues to develop during adolescence and is more vulnerable to parental factors [9]. Parental empathy toward the child is just a representative manifestation of how parents treat their children’s emotions in daily interactions [29].

Recent empirical studies have supported the relationship between parental empathy and children’s empathy. For example, cross-sectional studies and longitudinal studies in China have found a positive relationship between parental empathy and their children’s empathy ability, regardless of how the parental empathy was perceived by the child or parental self-reports [44]. However, these studies did not distinguish the different empathic components of the parent–child dyad. Thus, how different components of parental empathy and children’s own empathy ability are inter-related is still hard to clarify. In addition, a previous study showed that Chinese children and adolescents with higher-cognitive-empathy parents also show stronger social competence, which in turn leads to less emotional and behavioral problems [32]. Other-oriented empathy could be regarded as a typical indicator of social competence. This indirect but noteworthy evidence supports the positive relationship between parental cognitive empathy and adolescent other-oriented empathy. In addition, a previous study conducted in Argentina found that parents’ own cognitive empathy ability is a precursor to their children’s cognitive empathy and children’s concern for others’ emotions [45]. However, this study only focused on the general parental empathy ability. Considering that parental empathy toward the child occurs specifically in a family context, it can be different from parents’ general empathy ability [29,33]. Therefore, the potential positive relationship between parental cognitive empathy toward the adolescent child and the adolescent’s own other-oriented empathy ability still needs to be proved.

It is still worthy to investigate the role of different components of parental empathy in the mediation relationship between parental burnout and adolescent children’s empathy ability as well as behavioral development. In line with emotion-socialization theory, children may learn empathy from their parents. In this way, parental other-oriented empathy toward their children may be a precursor to adolescents’ other-oriented empathy ability and to self-oriented empathy.

Altogether, the mentioned theoretical model of the socialization of emotion and those research findings suggest that adolescents’ other-oriented empathy ability may serve as a sequential mediator going after parental cognitive empathy in the relationship between parental burnout and adolescent prosocial behavior. The second hypothesis was as follows:

**H2.**  *Parental burnout would positively predict adolescent prosocial behavior through the sequential mediating effects of parental cognitive empathy and adolescents’ other-oriented empathy ability.*

### 1.3. The Scope of this Study

The present study sought to examine a sequential mediation model to fill the gap in understanding the effect of parental burnout and adolescent prosocial behavior. Specifically, this study explored the relationship between parental burnout and adolescent prosocial behavior and the mediating mechanisms of parental cognitive empathy and adolescent other-oriented empathy. Based on theoretical and empirical evidence, the first hypothesis is that parental burnout would negatively predict adolescent prosocial behavior through the parental cognitive empathy ability. Second, the current study hypothesized that parental cognitive empathy and adolescents’ other-oriented empathy ability (adolescent cognitive empathy and empathic concern) sequentially would mediate the relationship between parental burnout and adolescent prosocial behavior. In other words, parental burnout would positively predict adolescent prosocial behavior through the sequential mediating effects of parental cognitive empathy and adolescents’ other-oriented empathy ability.

## 2. Materials and Methods

### 2.1. Participants

A total of 545 parent–adolescent dyads from two middle schools in a southeastern province of China were recruited for this study. This study was approved by the Ethics Committee of the first author’s university. Both adolescent students and their parents signed informed consent forms prior to the study. Dyadic data from youth and their parents were collected via online questionnaires. All the adolescents filled out questionnaires in quiet computer rooms at their school without receiving others’ help and placed their student numbers on the questionnaires, whereas the parents finished the online questionnaires at home. Both adolescents and their parents were free to withdraw from the study at any time. They filled in their child’s student number and we used it to match the child’s data. The adolescent students filled out questionnaires regarding empathy, prosocial behavior, and demographics, and the parents filled out questionnaires on parental burnout, parental empathy toward their children, and demographics. In addition, the social desirability scale was completed by both adolescents and their parents. Filling out the questionnaire took about 30 min for each participant.

After excluding 57 parent–adolescent dyads that did not complete the questionnaires carefully, determined by examining their answers to detecting questions, 488 parent–adolescent dyads were included in this study (for adolescents: *N* = 488, 45.7% males, 54.3% females; for parents: *N* = 488, 36.5% fathers, 63.5% mothers). The mean age of adolescents was 15.28 years, *SD* = 1.67 years, ranging from 12 to 19 years. The mean age of parents was 41.30 years, *SD* = 3.79 years, ranging from 32 to 49 years.

### 2.2. Measures

#### 2.2.1. Parental Burnout

To assess parental burnout, we used the Parental Burnout Assessment (PBA), which was developed by Roskam and his colleagues [11]. This 23-parent-self-reported-items questionnaire consisted of four subscales: exhaustion in parental role (9 items, e.g., “I have the sense that I’m really worn out as a parent.”); contrast in parental self (6 items, e.g., “I’m no longer proud of myself as a parent.”); feelings of being fed up (5 items, e.g., “I can’t take being a parent any more.”); and emotional distancing (3 items, e.g., “I’m no longer able to show my child(ren) how much I love them.”). The PBA items were rated on a 7-point Likert scale from 0 (never) to 6 (every day). Since parental burnout is a multidimensional latent variable, the mean score of each subscale was regarded as the observed indicators in the following structural equation model analysis. The internal consistency in the current sample (Cronbach’s α) was 0.92 for the global score, 0.91 for exhaustion in parental role, 0.76 for contrast in parental self, 0.82 for feelings of being fed up, and 0.62 for emotional distancing. The CFA results of the scale in the current study based on the original structure showed good validity (*x*^2^/*df* = 2.82, TLI = 0.93, RMSEA = 0.06, CFI = 0.92).

#### 2.2.2. Parental Empathy

Parental cognitive empathy and parental affective empathy were measured by using the Parent Empathy Measure (PEM) [29]. Adapted from mature empathy scales such as the Basic Empathy Scale (BES; Jolliffe and Farrington, 2006), this measure comprised 25 parent self-report items to create a specific indicator to represent how parents responded to their adolescent child’s emotions in the parent–child relationship [29]. The measure included two dimensions: parental cognitive empathy (14 items, e.g., “I can tell when my child is happy about something.”) and parental affective empathy (11 items, e.g., “My child’s emotions affect how I feel.”). Parents rated these items from 1 (not at all true) to 5 (very true). The internal consistency (Cronbach’s α) of the parental cognitive empathy and the parental affective empathy was 0.85 and 0.75, respectively. A CFA suggested that the two-factor model fitted well with the data, *x*^2^/*df* = 2.42, TLI = 0.92, RMSEA = 0.07, CFI = 0.91.

#### 2.2.3. Adolescent Empathy

Three components of adolescent empathy were assessed by three subscales of a validated Chinese version [46] of the Interpersonal Reactivity Index (IRI) [41], and each dimension included seven items. According to most previous research in the field of empathy [39,47], the perspective-taking subscale was used as an index of adolescent cognitive empathy (e.g., “When I’m upset at someone, I usually try to ‘put myself in his shoes’ for a while.”), the empathic concern subscale was used as an index of adolescent empathic concern (e.g., “I often have tender, concerned feelings for people less fortunate than me.”), and these two subscales also indicated adolescents’ other-oriented facets of empathy; while the personal-distress subscale was used as an index of adolescent affective empathy (e.g., “When I see someone who badly needs help in an emergency, I go to pieces.”), this subscale also indicated adolescents’ self-oriented facets of empathy. Adolescents indicated the degree to which they agreed or disagreed with a series of statements using a 5-point Likert scale from 1 (strongly disagree) to 5 (strongly agree). Cronbach’s α in the current study was 0.66 for personal distress, 0.65 for empathic concern, and 0.70 for perspective taking. The CFA results of the scale in the current study showed good validity (*x*^2^/*df* = 3.62, TLI = 0.93, RMSEA = 0.06, CFI = 0.92).

#### 2.2.4. Prosocial Behavior of Adolescents

To assess the prosocial behavior of adolescents, we used the compliant prosocial behavior subscale from the Chinese version of the Prosocial Tendencies Measure (PTM) [48], which was revised from Carlo and Randall’s version [49]. This subscale was chosen because it examines adolescents’ prosocial reactions when the individual expresses his or her emotions and needs, and this situation was both natural and typical in everyday life (e.g., “When people ask me to help them, I do not hesitate”). It includes five 7-point items (1 = does not describe me at all to 5 = describes me greatly, Cronbach’s α = 0.83). The CFA results of the scale in the current study based on the original structure showed good validity (*x*^2^/*df* = 2.44, TLI = 0.92, RMSEA = 0.05, CFI = 0.94).

#### 2.2.5. Social Desirability of Adolescents and Parents

Because adolescent and parent self-reported measures may be influenced to some extent by social desirability, the present study included the social desirability of adolescents and parents as control variables measured by the Chinese short version of the Crowne and Marlowe’s social desirability scale. Adapted from Reynolds (1982), this 13-item true–false questionnaire was created to determine the extent to which individuals adhere to the norms of society in their responses [50] (e.g., “No matter who I’m talking to, I’m always a good listener”), and a higher score indicated a greater tendency to characterize oneself positively. Cronbach’s α was 0.63 for the parental social desirability scale and 0.67 for the adolescent social desirability scale. The CFA results of the scale in the current study based on the original structure showed good validity (for parents reporting: *x*^2^/*df* = 2.78, *TLI* = 0.92, *RMSEA* = 0.05, *CFI* = 0.91; for adolescents reporting: *x*^2^/*df* = 3.19, TLI = 0.93, RMSEA = 0.06, CFI = 0.92).

#### 2.2.6. Family Socioeconomic Status

We asked adolescents to provide the number of books in their home and considered the response as the indicator of the family’s socioeconomic status (1 = less than 20 books to 5 = more than 200 books). This item has been used in many studies, including the Chinese PISA study [51]. It has shown a good correlation with the parents’ income and educational level, suggesting that it could be used as an accurate indicator of a family’s socioeconomic status. In the present study, family socioeconomic status was considered as a control variable [52,53].

### 2.3. Data Analyses

The data in the current study were analyzed with SPSS 22.0 and Mplus 7.4. Three sequential mediation models with latent variables were constructed according to the procedure suggested in previous studies [54]. For the multidimensional latent variable (parental burnout), the mean score of each factor was regarded as its observed indicators. Except for parental burnout, other unidimensional variables were regarded as the observed indicators.

In the three models, parental burnout was the antecedent variable and adolescent prosocial behavior was the consequential variable. For the first two models, parental cognitive empathy was the first mediating factor and adolescent cognitive empathy (first model) and adolescent empathy concern (second model) were the second mediating factors. For the third model, parental affective empathy was the first mediating factor and adolescent affective empathy was the second mediating factor.

In the mediation models of the current study, we controlled for sex, age, and social desirability for both adolescents and their parents, as well as the family’s socioeconomic status as reported by the adolescents as mentioned above. In addition, considering the significant correlation between parental affective empathy and cognitive empathy, we controlled the parental affective empathy in the first two models involving parental cognitive empathy, while in the third model involving parental affective empathy, we controlled the parental cognitive empathy.

**Figure 1:**
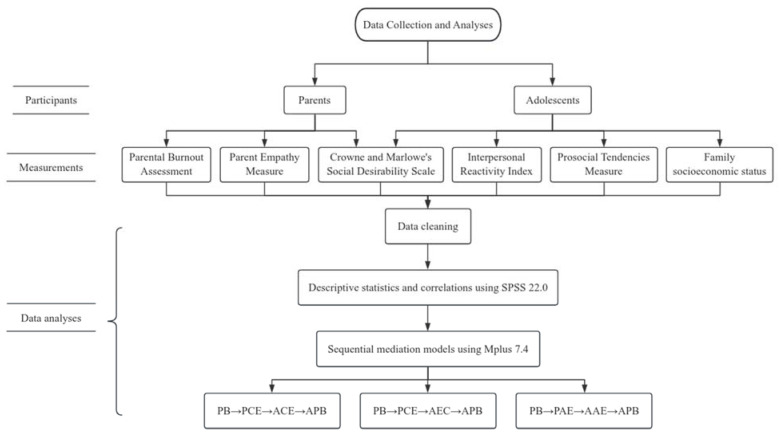
The flowchart for the current research technique. Note: PB = parental burnout, PCE = parental cognitive empathy, PAE = parental affective empathy, AAE = adolescent affective empathy, AEC = adolescent empathic concern, ACE = adolescent cognitive empathy, and APB = adolescent prosocial behavior.

A flowchart to illustrate the research technique of the current study is presented in Figure 1.

## 3. Results

### 3.1. Descriptive Results

Table 1 shows the means, SDs, and zero-order correlations for all research variables. Parental burnout was negatively associated with parental cognitive empathy and positively associated with parental affective empathy according to bivariate correlations across important variables. In addition, parental cognitive empathy was positively associated with adolescent empathic concern, cognitive empathy, and prosocial behavior and negatively associated with adolescent affective empathy. The adolescents’ other-oriented empathy ability (cognitive empathy and empathic concern) was positively associated with prosocial behavior.

### 3.2. Results of the Proposed Sequential Mediation Models

**Figure 2:**
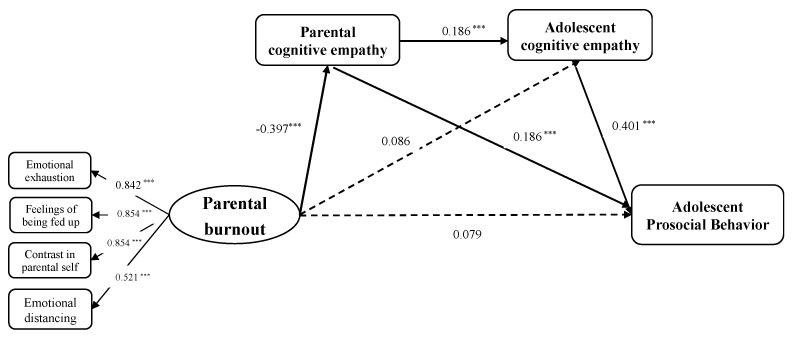
The sequential mediation model between parental burnout and adolescent prosocial behavior through parental cognitive empathy and adolescent cognitive empathy. Standardized coefficients are presented. Covariates were included in the model but are not presented for simplicity. Note: *** *p* < 0.001.

The three proposed sequential mediation models were tested. In the first model, the current study examined the mediating effect of parental cognitive empathy in the relationship between parental burnout and adolescent prosocial behavior. The results showed that the pathway from parental burnout to adolescent prosocial behavior through parental cognitive empathy was significant in this model. Furthermore, this study used the sequential mediation model to test whether parental burnout predicts adolescent prosocial behavior through parental cognitive empathy and adolescent cognitive empathy. This model demonstrated a sufficient goodness of fit to the data (*χ^2^* = 93.656, *df* = 43, CFI = 0.960, TLI = 0.928, SRMR = 0.049, RMSEA = 0.049). Figure 2 displays standardized parameter estimates of the sequential mediation model. The bias-corrected bootstrap procedure was used to test the significance of the mediating effects. The mediating effects and the 95% confidence intervals are presented in Table 2. Specifically, parental burnout was related to lower parental cognitive empathy and lower adolescent cognitive empathy sequentially and was finally linked to lower adolescent prosocial behavior. Detailed results of the multiple regression analysis of the indirect effect are presented in Table A1 of Appendix A.

**Figure 3:**
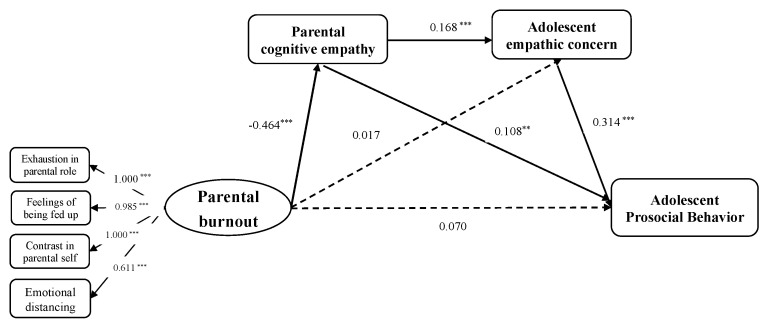
The sequential mediation model between parental burnout and adolescent prosocial behavior through parental cognitive empathy and adolescent empathic concern. Standardized coefficients are presented. Covariates were included in the model but are not presented for simplicity. Note: ** *p* < 0.01 and *** *p* < 0.001.

In the second model, the current study examined the mediating effects of parental cognitive empathy and adolescent empathic concern in the relationship between parental burnout and adolescent prosocial behavior. First, the results showed that the pathway from parental burnout to adolescent prosocial behavior through parental cognitive empathy was also significant in this model. Second, the proposed sequential mediation model demonstrated a sufficient goodness of fit to the data (*χ^2^* = 90.162, *df* = 43, CFI = 0.962, TLI = 0.931, SRMR = 0.048, RMSEA = 0.047). Figure 3 displays standardized parameter estimates of the sequential mediation model. The mediating effects and the 95% confidence intervals are presented in Table 2. Specifically, parental burnout was related to lower parental cognitive empathy and lower adolescent empathic concern sequentially and was finally linked to lower adolescent prosocial behavior. Detailed results of a multiple regression analysis of the indirect effects are presented in Table A2 of Appendix B.

**Figure 4:**
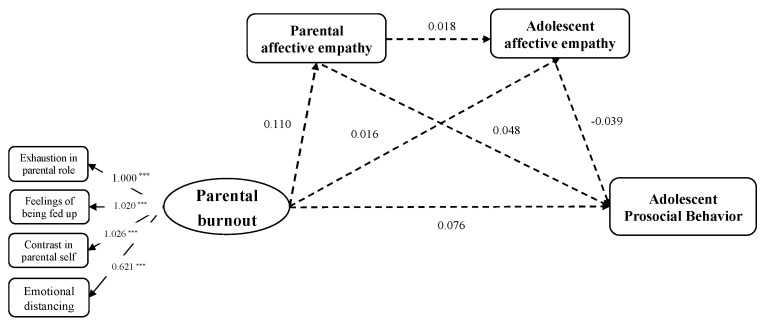
The insignificant sequential mediation model between parental burnout and adolescent prosocial behavior through parental affective empathy and adolescent affective empathy. Standardized coefficients are presented. Covariates were included in the model but are not presented for simplicity. *** *p* < 0.001.

In the third model, the results showed that the proposed sequential mediation model demonstrated a sufficient goodness of fit to the data (*χ^2^* = 162.205, *df* = 43, CFI = 0.902, TLI = 0.824, SRMR = 0.081, RMSEA = 0.075). Figure 4 displays standardized parameter estimates of the sequential mediation model, showing that all the indirect pathways and indirect effects were not significant. Detailed results of the multiple regression analysis of the indirect effect are presented in Table A3 of Appendix C.

In addition, to test the direct effect of parental burnout on adolescent prosocial behavior, a structural equation model was used, and the fit index (*χ^2^* = 73.490, *df* = 33, CFI = 0.96, TLI = 0.946, SRMR = 0.055, RMSEA = 0.050) showed a good model fit, while the results showed that the direct relationship between parental burnout and adolescent prosocial behavior was not significant. Detailed results of the multiple regression analysis of the direct effect are presented in Table A4 of Appendix D.

## 4. Discussion

The present study used a parent–adolescent matching design to evaluate the relationship between parental burnout and adolescent prosocial behavior. Mediation models were developed to better understand the pathways underlying the presumptive relationship based on the theoretical model of the socialization of emotion. The results indicated that parental burnout had a negative effect on adolescent-reported prosocial behavior through two pathways after controlling for demographic covariates and social desirability: one was that parental burnout is related to lower adolescent prosocial behaviors by impairing parental cognitive empathy, which entails the parents’ understanding of the child’s emotions and the reasons behind them, and the other was that parental burnout is linked to lower parental cognitive empathy and lower adolescent children’s other-oriented empathy sequentially (adolescent cognitive empathy and empathic concern as the sequential mediator, respectively), which in turn is associated with lower prosocial behaviors. Considering that previous studies have mainly focused on the antecedent variance of parental burnout, as well as its negative impacts on parents themselves and parenting behaviors [19,21,23], the current findings have advanced the understanding of how parental burnout, a family environmental factor, is associated with adolescent positive functioning through parental responses to their child’s emotions and the adolescents’ individual social-emotional ability.

### 4.1. The Crucial Mediating Role of Parental Cognitive Empathy on Their Children

Consistent with the first hypothesis in the introduction, parental cognitive empathy is a crucial factor mediating the relationship between parental burnout and adolescent prosocial behavioral outcomes. In other words, the negative effect of burnout on prosocial behavior must be activated through cognitive empathy. Specifically, parents with higher levels of burnout tended to be less cognitively empathic, which in turn reduced their children’s prosocial behaviors toward others.

To begin with, previous longitudinal studies conducted in China have shown a similar mediating pattern, suggesting that mothers that reported higher burnout in parenting performed more maternal hostility and less autonomy support, which result in poor mental health and more problematic behaviors in adolescents [13,28]. However, these two studies found that parenting style or behaviors are the mechanisms in the relationship between parental burnout and adolescent developmental outcomes. The current study extended previous findings, suggesting that parental reaction to their children’s emotions is another mediator. Taken together, these studies also extended most of the previous findings on the negative effects of parental burnout on parents themselves [35] by revealing how chronic and overwhelming negative emotional states are associated with the parental emotion-related interaction with children, and then with adolescent social behavioral development. As Hajal and Paley mentioned in the revised theoretical model of emotional socialization, parental emotions may affect their reaction to their children’s emotions, which in turn leads to emotional-related behavioral outcomes [24].

In addition, the present study suggested that parental burnout was inversely related to parental cognitive empathy in the first step of the mediating pathway. In line with the relationship, a previous study found that higher parental burnout leads to more parental violence and hostility as well as less supporting behaviors [13,28,35]. Combining the results of the current study, those maladaptive parental behaviors may be due to their failure to cognitively process adolescent children’s emotions. In addition, previous studies have also found that cognitive function is impaired after a long period of exposure to stress [35,36,55], while a significant improvement in cognitive function has been found after stress was regulated [56]. Taking that parental burnout results from chronic stress, its impairment in terms of parental cognitive empathy is consistent with the previous study, while, in line with this, parental affective empathy, which depends on the automatic processing of emotions [34], has not been destroyed by parental burnout.

As a consequence, the lower parental cognitive empathy toward their children results from parental burnout, in turn, is negatively associated with adolescent children’s prosocial behaviors. These findings are in line with the supplemented theoretical model of emotional socialization in a recent review, claiming that parents’ own emotions and their regulation are also critical to child emotion-socialization outcomes [24]. The mediating path found in the current research suggests that parental cognitive empathy plays a vital role in emotional socialization. Children learn to behave appropriately when confronted with others’ needs or emotions from how their emotions have been treated by their parents in a family context [8]. Specifically, burned-out parents’ cognitive way of reacting to their children’s emotions may be generalized by adolescent children to their social interactions, and then they perform less other-oriented social behaviors in daily life. This is consistent with previous findings that children and adolescents raised by indifferent and neglectful parents display less altruistic behaviors toward others [57].

### 4.2. The Sequential Mediating Roles of Adolescent Cognitive Empathy and Empathic Concern

Consistent with the second hypothesis in the introduction, parental cognitive empathy toward their children is positively related to adolescents’ own cognitive empathy and empathic concern ability, suggesting that parental cognitive empathy is the first mediator and that two facets of adolescents’ own other-oriented empathy (adolescent cognitive empathy and empathic concern) are the sequential mediators. To be specific, higher parental burnout predicted lower parental cognitive empathy and then led to lower adolescent cognitive empathy and empathic concern ability, which finally impaired their prosocial behavior.

It is also noteworthy that parental perceptions of exhausted emotions only may not be enough to impair adolescent children’s prosocial outcomes; the less prosocial behaviors may derive from the emotional socialization process, in which the children learn empathic patterns during communication with their parents. This sequential transmissional pattern of other-oriented empathy in parent–child dyads is also in line with previous studies. The results of a parent–child dyadic designed study showed that parental cognitive aspects of empathy are positively related to children’s other-oriented empathy [58]. The emotional socialization process was also found in a previous study, suggesting that parental cognitive empathy was positively associated with children’s emotion-related social competence, which in turn negatively affected children’s behavioral problems [32]. The relationship between adolescent other-oriented empathy and their prosocial behavior was consistent with the empathy–altruism hypothesis [30], which states that other-oriented empathic feelings for a person in need evoke prosocial motivation to meet that person’s needs. Accordingly, previous longitudinal studies provided evidence that cognitive empathy and the empathic concern of adolescents stably predicted their prosocial behavior [40,41].

The current finding was also in line with the theoretical model of the socialization of emotion [8,24], revealing that parental responses to children’s emotions resulted from the parents’ own emotional experiences set as an example for adolescent children when they are confronted with the misfortune of others. Children’s own emotional-related ability (e.g., their understanding of emotion and regulation and their affective stance toward emotions and oneself) may play as a mediator between parental reactions to children’s emotions and the social behavioral outcome of their children. Adolescent cognitive empathy is an indicator of their understanding of others’ emotions, and empathic concern reflects their emotion-related motivation to pay attention to others’ emotions [34].

The results showed that only cognitive empathy and adolescents’ other-oriented empathy ability (cognitive empathy and empathic concern) served the sequential mediating role, while the sequential mediating path from parental burnout to adolescent prosocial behavior through parental affective empathy and adolescents’ affective empathy ability was not significant. It is important to note that parental affective empathy is not associated with the outcome variables. Firstly, the developmental characteristics of affective empathy may help to explain the nonsignificant correlation between parental affective empathy toward their children and adolescents’ affective empathy ability. A metanalysis and longitudinal studies have found that affective empathy begins very early in one’s life and reaches a mature and stable level in adolescence [59,60]. Hence, it is less likely to be impacted by environmental factors, while other-oriented empathy is still under development during adolescence [59,60] and may be more sensitive to parental factors during interactions. Secondly, the nonsignificant relationship between parental affective empathy and adolescent prosocial behavior may be due to the different parental behaviors that resulted from distinct parental empathy components. Previous findings have suggested that parents who were more likely to have alternative feelings toward their children (were high in parental affective empathy) showed less children-oriented caring behavior [61], whereas those parents who understood their adolescent children’s emotions accurately (were high in parental cognitive empathy) would perform more supporting parental behaviors [37], which is positively associated with adolescent prosocial behavior [57]. In this way, the distinct function of parental cognitive empathy and parental affective empathy may be reasonable. This explanation is also in line with the theoretical model of emotion socialization, stating that parents provide emotion-related scaffold to their children that further impact their children’s behaviors when dealing with others’ emotions [24]. Moreover, the nonsignificant relationship between adolescent affective empathy and prosocial behavior could be explained from the perspective of an underlying motive of affective empathy. Previous work has found that the affective empathy ability has a positive relationship with motives and will make individuals escape from others’ suffering when they are in need [62]. In addition, parental burnout only impaired parental cognitive empathy. This may be because the overwhelming stress of burned-out parents destroys their cognitive resources to process their children’s emotions [56], while the emotional contagion is a bottom-up automatic response that does not depend on cognitive resources.

The current study made the pathway more nuanced by clearly differentiating parental self-oriented reactions (parental affective empathy) and other-oriented reactions with their children (parental cognitive empathy), as well as their children’s self-oriented empathy ability (adolescent affective empathy) and other-oriented empathy ability (adolescent cognitive empathy and empathic concern). Although the indirect effects are minor, the sequential mediating results indeed have both theoretical and empirical foundations and deserve further investigation in the future.

### 4.3. Implications

The present study has both theoretical and practical implications. From a theoretical perspective, firstly, the present study is the first to confirm that parental burnout has an indirect effect on adolescent prosocial behavior through parental empathy and adolescent empathy. This study extends previous research on the understanding of how parental burnout decreases adolescent prosocial behavior. These unique effects of parental burnout on adolescent prosocial behavior deserve greater attention in further studies. Secondly, the research focuses on different facets of parental and adolescent empathy, which clarifies the mixed results of previous studies and provides a more nuanced understanding of the relationship between parental burnout and adolescent prosocial behavior. These findings highlight that future studies should distinguish the effects of different facets of empathy on adolescent socialization.

From a practical perspective, firstly, the results show that parental burnout is a risk factor for adolescent prosocial behavior. This implies that parents are supposed to learn some of the characteristics of their children during puberty. It also highlights that educators should teach parents some parenting skills to communicate with their adolescents effectively, which may reduce parental burnout. Secondly, this study showed that both adolescents’ social emotion ability and parental emotions and emotional reactions to their children contribute to adolescent prosocial behavior. This means the whole family system should be taken into consideration when designing interventional programs for adolescent prosocial behavior. Specifically, it is necessary to consider that the intervention targets the improvement in cognitive empathy and motivation for both parents and adolescents. For instance, the Family Check-Up (FCU) [63], a family-centered intervention, can be used to facilitate parents’ concern and sensitivity to their child’s emotions and behavior. Another program named Families Overcoming Under Stress (FOCUS) [64] is also worth learning from, for it involves both parents and children to develop a common language for talking about their own and each other’s emotions when targeting children’s adjustment.

### 4.4. Limitations

Despite the contributions of this study, it also had some limitations. First, the present study is built in cross-section. Although the mediation models in the current study were based on theoretical underpinnings and empirical investigations, the sequential mediating relationship must be interpreted with caution from a causal perspective. Second, the variables involved in the current study were all self-reported by parents or adolescents. Even though the social desirability of adolescents and parents was controlled rigidly, laboratory observation methods should be considered in the future. Third, the relatively small effect size of the sequential mediation in the current study had to be highly regarded. On one hand, it may be due to the small sample size, and on the other hand, it may indicate the existence of other potential mediating variables in the relationship between parental burnout and adolescent prosocial behavior. However, the current study only focused on empathy, leaving other mediators unexcavated. Fourth, the study’s generalizability is limited because it focuses only on a Chinese sample. As a collectivist society, China places a strong emphasis on family ties [65]. One must therefore exercise caution when extrapolating current conclusions to different cultural contexts.

### 4.5. Future Research Directions

Firstly, future longitudinal studies are necessary. Considering the limitations of cross-sectional designs in revealing causal relationships, longitudinal methods will provide more consolidated evidence to reveal the mechanisms involved in the relationship between parental burnout and adolescents’ prosocial behavior. Secondly, it is necessary for future studies to verify the sequential mediating effect through higher ecological validity measurements in a larger sample. For example, parental empathy could be examined by experimental simulation in more natural parent–child interaction situations (like parental mimicking and understanding of their children’s emotions) [66]. Similarly, adolescent empathy and prosocial behavior could also be measured by using an experimental paradigm with higher ecological validity. Moreover, it is essential to explore other mediating pathways. According to Eisenberg’s theoretical model of emotion socialization, both the expression of emotions and parental reactions to children’s emotions are proposed to have an impact on children’s emotional development [8]. Future studies could also validate whether parental emotion expression is the mediator between parental burnout and adolescent prosocial outcomes. Additionally, the generalizability of our findings should be further validated with samples from other countries. Future research should involve adolescents from many cultural groups and investigate whether comparable results can be produced in nations with an individualistic culture.

## 5. Conclusions

Parents who experience ongoing stress from parenting may be vulnerable to parental burnout, which can negatively affect the social development of their children. But no previous study has looked at the long-term effects of parental burnout on children’s prosocial behavior. Using a parent–adolescent dyadic design, the current study extended prior works and investigated the sequential mediation model through which parental burnout was linked to adolescent prosocial behavior. Specifically, parental burnout predicted adolescent prosocial behavior negatively through the mediating effect of parental cognitive empathy and the sequential mediating effect of adolescent other-oriented empathy. Taken together, based on the affective organization of the parenting framework, the theoretical model of the socialization of emotion, and the empathy–altruism hypothesis, the current findings improved the understanding of how parental burnout is associated with adolescent prosocial behavior through the emotion-socialization process. To explore this pathway of influence more deeply, qualitative methodologies are required in future studies. It is necessary to conduct interviews from the perspective of adolescents to address the specific manifestations of parental burnout in parenting and parent–child interactions, and how it affects them when their parents are burned out. In addition to the adolescents’ perspectives, organizing interviews from the parents’ perspectives on why parental burnout occurs and observing the interactions between burned-out parents and their children would deepen the findings of the current study.

## Figures and Tables

**Table 1:** Descriptive statistics and correlations of the variables in the current study.

	1	2	3	4	5	6	7	8	9	10	11	12	13	14
A-Gen	—													
2. A-Age	−0.038	—												
3. A-Sd	−0.048	0.014	—											
4. P-Gen	0.031	0.196 **	−0.038	—										
5. P-Age	−0.081	0.272 **	−0.025	−0.161 ***	—									
6. P-Sd	−0.048	0.014	0.218 ***	0.038	−0.025	—								
7. FSES	−0.074	0.099 *	−0.055	−0.037	0.090 *	−0.055	—							
8. PB	0.045	−0.166 ***	−0.018	0.103 *	0.031	−0.018	−0.048	—						
9. P-AE	0.054	0.173 ***	−0.160 ***	−0.063	0.123 **	−0.194 ***	−0.011	0.138 **	—					
10. P-CE	0.006	−0.069	0.021	−0.008	−0.058	−0.013	0.169 ***	−0.382 ***	−0.287 ***	—				
11. A-AE	0.195 ***	−0.033	−0.197 ***	−0.047	0.048	0.056	−0.055	0.072	0.058	−0.111 *	—			
12. A-EC	0.164 ***	0.011	−0.150 ***	−0.052	−0.022	−0.072	−0.012	−0.047	−0.068	0.161 ***	0.322 ***	—		
13. A-CE	0.079	0.122 **	−0.076	0.035	0.033	−0.057	0.045	−0.097	−0.021	0.187 ***	−0.099 *	0.511 ***	—	
14. A-Pb	0.006	0.045	−0.296 ***	−0.022	−0.008	−0.089 *	−0.015	0.005	0.051	0.107 *	0.010	0.354 ***	0.421 ***	—
M	—	15.28	1.52	—	41.30	1.59	—	1.69	2.88	3.70	3.35	3.66	3.62	3.69
(SD)	—	1.67	0.25	—	3.79	0.24	—	0.79	0.53	0.54	0.58	0.55	0.53	0.63

Note: * *p* < 0.05, ** *p* < 0.01, and *** *p* < 0.001. A-Gen = sex of adolescents (coded as 0 = male and 1 = female), A-Age = age of adolescents, P-Gen = gender of parents (coded as 0 = male and 1 = female), P-Age = age of parents, A-Sd = adolescent social desirability, P-Sd = parental social desirability, FSES = family socioeconomic status, PB = parental burnout, P-AE = parental affective empathy, P-CE = parental cognitive empathy, A-AE = adolescent affective empathy, A-EC = adolescent empathic concern, A-CE = adolescent cognitive empathy, and A-Pb = adolescent prosocial behavior.

**Table 2:** The bootstrapping analysis of the mediation effects.

	Pathways	Indirect Effects	*SE*	95% CI	
				Lower	Upper
Model 1	parental burnout → parental cognitive empathy → adolescent prosocial behavior	−0.034	0.018	−0.063	−0.006
	parental burnout → adolescent cognitive empathy → adolescent prosocial behavior	−0.015	0.019	−0.047	0.016
	parental burnout → parental cognitive empathy → adolescent cognitive empathy → adolescent prosocial behavior	−0.030	0.009	−0.048	−0.016
Model 2	parental burnout → parental cognitive empathy → adolescent prosocial behavior	−0.043	0.019	−0.076	−0.014
	parental burnout → adolescent empathic concern → adolescent prosocial behavior	0.005	0.016	−0.020	0.030
	parental burnout → parental cognitive empathy → adolescent empathic concern → adolescent prosocial behavior	−0.021	0.007	−0.036	−0.011
Model 3	parental burnout → parental affective empathy → adolescent prosocial behavior	0.004	0.007	−0.001	0.028
	parental burnout → adolescent affective empathy → adolescent prosocial behavior	−0.001	0.004	−0.009	0.003
	parental burnout → parental affective empathy → adolescent affective empathy → adolescent prosocial behavior	<0.001	<0.001	−0.002	<0.001

## Data Availability

The data presented in this study are available upon request from the corresponding author.

## Appendix A

**Table A1:** Result of multiple regression analysis of indirect effect in model 1.

Dependent Variable	Independent Variable	Standardized Estimate			Model Fit
		β	*t*	95% CI	R^2^
P-CE	P-GEN	0.01	0.13	[−0.06, 0.08]	0.22
	A-GEN	0.03	0.75	[−0.04, 0.10]	
	P-AGE	−0.02	−0.40	[−0.09, 0.05]	
	A-AGE	−0.12	−2.68 **	[−0.19, −0.05]	
	P-SD	−0.04	−0.88	[−0.11, 0.04]	
	A-SD	0.02	0.37	[−0.05, 0.09]	
	FSES	0.17	4.17 ***	[0.09, 0.24]	
	P-AE	−0.13	−2.94 **	[−0.21, −0.04]	
	PB	−0.40	−9.78 ***	[−0.46, −0.32]	
A-CE	P-GEN	0.07	1.47	[−0.01, 0.14]	0.07
	A-GEN	0.08	1.73	[0.003, 0.15]	
	P-AGE	0.02	0.46	[−0.05, 0.10]	
	A-AGE	0.14	2.80 **	[0.06, 0.22]	
	P-SD	−0.02	−0.30	[0.10, 0.06]	
	A-SD	−0.07	−1.66	[−0.14, 0.01]	
	FSES	0.00	−0.01	[−0.08, 0.07]	
	P-AE	0.01	0.20	[−0.07, 0.09]	
	PB	−0.04	−0.79	[−0.11, 0.04]	
	P-CE	0.19	3.79 ***	[0.11, 0.27]	
A-Pb	P-GEN	−0.06	−1.39	[−0.12, 0.01]	0.26
	A-GEN	−0.05	−1.21	[−0.12, 0.02]	
	P-AGE	−0.04	−0.96	[−0.11, 0.03]	
	A-AGE	0.01	0.27	[−0.07, 0.09]	
	P-SD	−0.01	0.16	[−0.08, 0.06]	
	A-SD	−0.27	−6.31 ***	[−0.34, −0.20]	
	FSES	−0.06	−1.44	[−0.14, 0.004]	
	P-AE	0.04	0.99	[−0.03, 0.12]	
	PB	0.08	1.56	[−0.01, 0.15]	
	P-CE	0.09	1.95	[0.01, 0.16]	
	A-CE	0.40	9.72 ***	[0.33, 0.47]	

Note: ** *p* < 0.01 and *** *p* < 0.001. A-GEN = sex of adolescents (coded as 0 = male and 1 = female), A-AGE = age of adolescents, P-GEN = gender of parents (coded as 0 = male and 1 = female), P-AGE = age of parents, A-SD = adolescent social desirability, P-SD = parental social desirability, FSES = family socioeconomic status, PB = parental burnout, P-AE = parental affective empathy, P-CE = parental cognitive empathy, A-CE = adolescent cognitive empathy, and A-Pb = adolescent prosocial behavior.

## Appendix B

**Table A2:** Result of multiple regression analysis of indirect effect in model 2.

Dependent Variable	Independent Variable	Standardized Estimate			Model Fit
		β	*t*	95% CI	R^2^
P-CE	P-GEN	0.01	0.12	[−0.05, 0.08]	0.22
	A-GEN	0.03	0.73	[−0.04, 0.10]	
	P-AGE	−0.02	−0.40	[−0.09, 0.05]	
	A-AGE	−0.12	−2.64 **	[−0.19, −0.05]	
	P-SD	−0.04	−0.85	[−0.11, 0.04]	
	A-SD	0.02	0.37	[−0.05, 0.09]	
	FSES	0.17	3.85 ***	[0.09, 0.24]	
	P-AE	−0.13	−2.53**	[−0.21, −0.04]	
	PB	−0.40	−9.86 ***	[−0.46, −0.32]	
A-EC	P-GEN	0.06	−1.35	[−0.14, 0.02]	0.08
	A-GEN	0.16	3.51 ***	[0.08, 0.23]	
	P-AGE	−0.02	−0.35	[−0.08, 0.06]	
	A-AGE	0.03	0.66	[0.04, 0.12]	
	P-SD	−0.04	−0.81	[−0.12, 0.05]	
	A-SD	−0.15	−3.31 **	[−0.23, −0.08]	
	FSES	−0.05	−0.99	[−0.12, 0.03]	
	P-AE	−0.05	−1.14	[−0.14, 0.02]	
	PB	0.01	0.39	[−0.07, 0.09]	
	P-CE	0.17	3.53 ***	[0.08, 0.24]	
A-Pb	P-GEN	−0.01	−0.23	[−0.07, 0.06]	0.22
	A-GEN	−0.07	−1.49	[−0.14, 0.01]	
	P-AGE	−0.03	−0.64	[−0.09, 0.04]	
	A-AGE	0.06	1.17	[−0.03, 0.14]	
	P-SD	<0.001	0.01	[−0.07, 0.07]	
	A-SD	−0.25	−5.64 ***	[−0.32, −0.17]	
	FSES	−0.05	−1.05	[−0.14, 0.02]	
	P-AE	0.07	1.37	[−0.01, 0.15]	
	PB	0.06	1.10	[−0.01, 0.15]	
	P-CE	0.11	2.33	[0.04, 0.19]	
	A-EC	0.31	6.43 ***	[−0.04, 0.14]	

Note: ** *p* < 0.01 and *** *p* < 0.001. A-GEN = sex of adolescents (coded as 0 = male and 1 = female), A-AGE = age of adolescents, P-GEN = gender of parents (coded as 0 = male and 1 = female), P-AGE = age of parents, A-SD = adolescent social desirability, P-SD = parental social desirability, FSES = family socioeconomic status, PB = parental burnout, P-AE = parental affective empathy, P-CE = parental cognitive empathy, A-EC = adolescent empathic concern, and A-Pb = adolescent prosocial behavior.

## Appendix C

**Table A3:** Result of multiple regression analysis of indirect effect in model 3.

Dependent Variable	Independent Variable	Standardized Estimate			Model Fit
		β	*t*	95% CI	R^2^
P-AE	P-GEN	−0.032	−0.71	[−0.10, 0.05]	0.11
	A-GEN	0.06	0.40	[−0.02, 0.13]	
	P-AGE	0.05	1.21	[−0.01, 0.13]	
	A-AGE	0.14	2.83 **	[0.06, 0.22]	
	P-SD	−0.15	−2.99	[−0.22, 0.06]	
	A-SD	−0.12	−2.79 **	[−0.20, −0.05]	
	FSES	−0.01	−0.27	[−0.09, 0.06]	
	P-CE	−0.14	−2.55 **	[−0.23, −0.04]	
	PB	0.09	1.17	[−0.02, 0.24]	
A-AE	P-GEN	−0.06	−1.43	[−0.14, 0.05]	0.10
	A-GEN	0.19	4.29 ***	[0.11, 0.26]	
	P-AGE	0.06	1.37	[−0.02, 0.13]	
	A-AGE	−0.04	−0.90	[−0.13, 0.03]	
	P-SD	0.11	2.37 *	[0.03, 0.18]	
	A-SD	−0.21	−5.34 ***	[−0.28, −0.15]	
	FSES	−0.03	−0.72	[−0.10, 0.04]	
	P-CE	−0.09	−1.78 *	[−0.18, −0.02]	
	PB	0.01	0.25	[−0.09, 0.09]	
	P-AE	0.02	0.42	[−0.05, 0.09]	
A-Pb	P-GEN	−0.03	−0.72	[−0.10, 0.04]	0.12
	A-GEN	−0.01	−0.23	[−0.09, 0.06]	
	P-AGE	−0.03	−0.68	[−0.11, 0.04]	
	A-AGE	0.06	1.29	[−0.02, 0.15]	
	P-SD	−0.009	−0.19	[−0.09, 0.06]	
	A-SD	−0.30	−6.58 ***	[−0.37, −0.22]	
	FSES	−0.06	−1.27	[−0.16, 0.10]	
	P-CE	0.15	3.26 **	[0.08, 0.23]	
	PB	0.06	1.18	[−0.03, 0.14]	
	P-AE	0.05	0.97	[−0.03, 0.13]	
	A-AE	−0.04	−0.84	[−0.04, 0.04]	

Note: * *p* < 0.05, ** *p* < 0.01, and *** *p* < 0.001. A-GEN = sex of adolescents (coded as 0 = male and 1 = female), A-AGE = age of adolescents, P-GEN = gender of parents (coded as 0 = male and 1 = female), P-AGE = age of parents, A-SD = adolescent social desirability, P-SD = parental social desirability, FSES = family socioeconomic status, PB = parental burnout, P-AE = parental affective empathy, P-CE = parental cognitive empathy, A-AE = adolescent affective empathy, and A-Pb = adolescent prosocial behavior.

## Appendix D

**Table A4:** Result of multiple regression analysis on direct effect of parental burnout on adolescent prosocial behavior.

Dependent Variable	Independent Variable	Standardized Estimate			Model Fit
		β	*t*	95%CI	R^2^
A-Pb	P-GEN	−0.03	−0.64	[−0.10, 0.04]	0.09
	A-GEN	−0.01	−0.25	[−0.09, 0.06]	
	P-AGE	−0.03	−0.75	[−0.11, 0.04]	
	A-AGE	0.05	1.04	[−0.03, 0.13]	
	P-SD	−0.02	−0.53	[−0.10, 0.04]	
	A-SD	−0.30	−6.76 ***	[−0.36, −0.22]	
	FSES	−0.04	−0.77	[−0.13, 0.04]	
	PB	0.01	0.10	[−0.09, 0.09]	

Note: *** *p* < 0.001. A-GEN = sex of adolescents (coded as 0 = male and 1 = female), A-AGE = age of adolescents, P-GEN = gender of parents (coded as 0 = male and 1 = female), P-AGE = age of parents, A-SD = adolescent social desirability, P-SD = parental social desirability, FSES = family socioeconomic status, PB = parental burnout, P-AE = parental affective empathy, P-CE = parental cognitive empathy, A-AE = adolescent affective empathy, A-EC = adolescent empathic concern, A-CE = adolescent cognitive empathy, and A-Pb = adolescent prosocial behavior.
